# Ancient Mitogenomes Provide New Insights into the Origin and Early Introduction of Chinese Domestic Donkeys

**DOI:** 10.3389/fgene.2021.759831

**Published:** 2021-10-15

**Authors:** Linying Wang, Guilian Sheng, Michaela Preick, Songmei Hu, Tao Deng, Ulrike H. Taron, Axel Barlow, Jiaming Hu, Bo Xiao, Guojiang Sun, Shiwen Song, Xindong Hou, Xulong Lai, Michael Hofreiter, Junxia Yuan

**Affiliations:** ^1^ Faculty of Materials Science and Chemistry, China University of Geosciences, Wuhan, China; ^2^ School of Environmental Studies, China University of Geosciences, Wuhan, China; ^3^ State Key Laboratory of Biogeology and Environmental Geology, China University of Geosciences, Wuhan, China; ^4^ Institute for Biochemistry and Biology, University of Potsdam, Potsdam, Germany; ^5^ Shaanxi Provincial Institute of Archaeology, Xi’an, China; ^6^ Key Laboratory of Vertebrate Evolution and Human Origins of Chinese Academy of Sciences, IVPP, Beijing, China; ^7^ School of Science and Technology, Nottingham Trent University, Nottingham, United Kingdom; ^8^ School of Earth Sciences, China University of Geosciences, Wuhan, China

**Keywords:** Chinese domestic donkeys, ancient DNA, mitochondrial genome, maternal lineage, divergence time

## Abstract

Both molecular data and archaeological evidence strongly support an African origin for the domestic donkey. Recent genetic studies further suggest that there were two distinct maternal lineages involved in its initial domestication. However, the exact introduction time and the dispersal process of domestic donkeys into ancient China are still unresolved. To address these questions, we retrieved three near-complete mitochondrial genomes from donkey specimens excavated from Gaoling County, Shaanxi Province, and Linxia Basin, Gansu Province, China, dated at 2,349-2,301, 469-311, and 2,160-2,004 cal. BP, respectively. Maximum-likelihood and Bayesian phylogenetic analyses reveal that the two older samples fall into the two different main lineages (i.e., clade Ⅰ and clade Ⅱ) of the domestic donkey, suggesting that the two donkey maternal lineages had been introduced into Midwestern China at least at the opening of Silk Road (approximately the first century BC). Bayesian analysis shows that the split of the two donkey maternal lineages is dated at 0.323 Ma (95% CI: 0.583–0.191 Ma) using root-tip dating calibrations based on near-complete mitogenomes, supporting the hypothesis that modern domestic donkeys go back to at least two independent domestication events. Moreover, Bayesian skyline plot analyses indicate an apparent female population increase between 5,000 and 2,500 years ago for clade I followed by a stable population size to the present day. In contrast, clade II keeps a relatively stable population size over the past 5,000 years. Overall, our study provides new insights into the early domestication history of Chinese domestic donkeys.

## Introduction

The domestication of the donkey (*Equus asinus*) is a vital event in human history, which played a significant role in the development of human civilization (Liu et al., 2010; Bai et al., 2017; Wang et al., 2020). Donkey deeply transformed ancient societies and land-based transport in Africa and Eurasia, contributed to the growth of the early Egyptian State, and allowed the development of mobile pastoralism and ancient overland trade routes (Denbow et al., 1993; Denham and Iriarte, 2007; Rossel et al., 2008; Kimura et al., 2010). The domestication of the donkey therefore probably indicates a major cultural shift away from sedentary, agrarian life-styles towards more migration and trade in ancient times (Beja-Pereira et al., 2004; Han et al., 2014). However, compared with the other domesticated species of the genus *Equus*, i.e., the horse, the domestic donkey is greatly underrepresented in the scientific literature (Blench, 2000; Lu et al., 2008; Ma et al., 2020). In the last decades with the promotion of agricultural mechanization and the rapid development of the transportation industry in modern society, the role of domestic donkeys as a means of transportation is decreasing and the number of donkeys has declined greatly (Xie, 1987). Despite these developments, currently donkeys still remain an essential means of transport for people living in mountain areas, deserts, and underdeveloped regions of the world (Blench, 2000; Smith and Pearson, 2005; Kimura et al., 2010; Ma et al., 2020).

Archeological evidence suggests an African origin for the donkey (Epstein, 1971; Clutton-Brock, 1992; Rossel et al., 2008). The earliest domestic donkey remains, 5,000-year-old ass skeletons, have been excavated from an early pharaonic mortuary complex at Abydos, Middle Egypt, which exhibit a range of osteopathologies consistent with load carrying (Rossel et al., 2008). However, it is often difficult to determine whether the remains from early phases of animal domestication originate from animals that have been domesticated or not (Peters and von der Driesc, 1997; Rossel et al., 2008). Compared with the horse, donkey remains are relatively rare in archaeological sites and are not easily distinguished from the former based on morphological characters alone (Han et al., 2014). Therefore, the available morphological evidence provides limited information about the timing and location of donkey domestication.

Mitochondrial and nuclear DNA have revealed that domestic donkeys originated from African wild asses (Ivankovic et al., 2002; Beja-Pereira et al., 2004; Kimura et al., 2010; Ma et al., 2020; Wang et al., 2020). Mitochondrial DNA studies showed that domestic donkeys harbored two distinct lineages (i.e., clade I and clade II). Clade I (Nubian lineage) contains domestic donkeys and the Nubian wild ass (*Equus africanus africanus*), while clade II (unknown origin) probably derived from a now extinct African wild ass population, which might have been close to the Somali wild ass (*Equus africanus somaliensis*) (Kimura et al., 2010, 2013; Ma et al., 2020). Wang et al. (2020) recently analyzed 126 modern domestic donkey nuclear genomes. Their *D-statistic* analysis showed an African domestication of donkeys, consistent with the results from mitochondrial DNA, and indicated its subsequent spread to Europe and Asia. In addition, the principal component analysis (PCA) suggested that domestic donkeys are divided into three main clusters on the nuclear level, i.e., a Tropical Africa cluster, a North Africa and Eurasia cluster and an Australia cluster. Wang et al. (2020) finally found that domestic donkeys showed reduced levels of Y chromosome variability, which might indicate a discordance of paternal and maternal histories of donkeys, similar to the domestic horse (Lindgren et al., 2004; Lippold et al., 2011).

The history of domestic donkey in China dates back more than 4,000 years (Zheng, 1980; Xie, 1987; Chen et al., 2010). According to literature records, domestic donkeys were bred in present-day Shache County, Xinjiang Uygur Autonomous Region, Northwestern China as early as in the Yin and Shang Dynasties (1,300-1,046 BC) (Yang and Hong, 1989). Regarding the origin of the Chinese domestic donkey, there are two main views: 1) Due to morphological similarities to Asian wild asses, e.g., in fur color, some researchers believed that Chinese domestic donkeys might have originated from Mongolian wild ass (*Equus hemionus*) (Xie, 1987; Liu et al., 2010). 2) In contrast, genetic studies suggested that Chinese domestic donkeys originate from African wild asses (Sun et al., 2007; Han et al., 2014; Ma et al., 2020). Wang et al. (2020) analyzed mitochondrial DNA and nuclear genomes of Chinese local donkey breeds and revealed that Chinese donkeys are closer to African wild asses than to Asian wild asses (*E. hemionus* and *Equus kiang*). So far, most molecular studies on Chinese domestic donkey focus on modern specimens. The only public report on genetic analyses of Chinese ancient donkeys has been presented by Han et al. (2014), but only mitochondrial DNA D-loop and cytochrome *b* gene fragments were obtained, with the dates of the analyzed samples ranging between 1,200–550 years before present (BP). Han et al. (2014) found that the ancient specimens represent both donkey mitochondrial maternal lineages, i.e., the Nubian lineage (clade Ⅰ) and the lineage of unknown origin (clade Ⅱ). Unfortunately, due to a lack of genetic information from earlier Chinese donkeys, we know little about the initial dispersal process of donkeys into China.

In this study, we retrieved three near-complete mitogenomes from archaeological donkey specimens excavated from Midwestern China, investigated the phylogenetic status of the analyzed individuals and estimated the divergence time of the two donkey lineages. We also carried out a Bayesian skyline plot (BSP) analysis to assess donkey population dynamics. Overall, our study provides new insights into the early domestication history of Chinese donkeys.

## Results and Discussion

**FIGURE 1:**
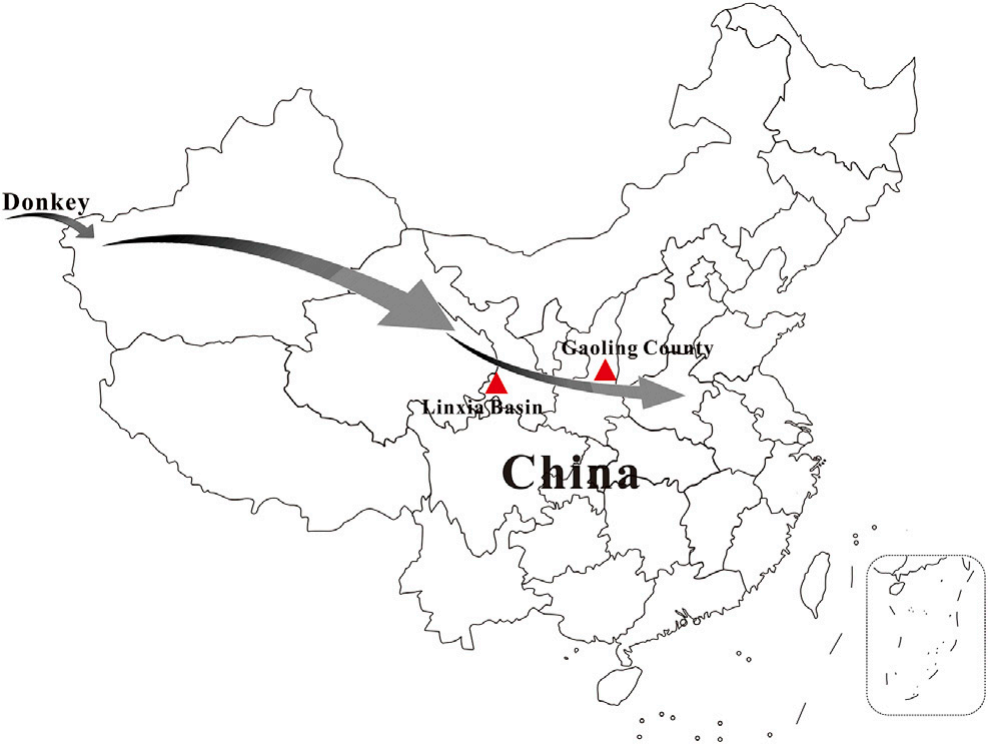
Sampling locations of Chinese ancient donkeys analyzed in this study. Sample LXH1 was excavated from Linxia Basin, specimens SG1 and SG3 were collected from Gaoling County. The arrows indicate possible dispersal route of domestic donkey to ancient China.

Three ancient donkey tooth samples are included in this study. Two specimens (SG1 and SG3) were excavated from Gaoling County, Shaanxi Province, China and one sample (LXH1) was collected from Linxia Basin, Gansu Province, China (Figure 1). ^14^C dating of the samples was performed by accelerator mass spectrometry (AMS) at the Archaeological Geochronology Laboratory of Peking University (PKUAMS, China). Calibration was done using IntCal13 (Reimer et al., 2013), yielding ages of 2,349-2,301 (SG1), 469-311 (SG3), and 2,160-2,004 (LXH1) cal. BP, respectively. Detailed information on the samples is listed in Supplementary Table S1. Using hybridization capture technology and an *E. asinus* mitogenome (GenBank No. X97337) as reference, we successfully retrieved three near-complete mitochondrial genomes from the analyzed samples with a mean depth of 79.5-, 37.2- and 36.8-fold, respectively (Supplementary Table S1). Mitochondrial DNA (mtDNA) fragments show damage patterns characteristic for ancient DNA (Briggs et al., 2007) (Supplementary Figure S1), supporting the obtained sequences as derived from authentic ancient DNA.

### Early Dispersal of Domestic Donkey to Ancient China

**FIGURE 2:**
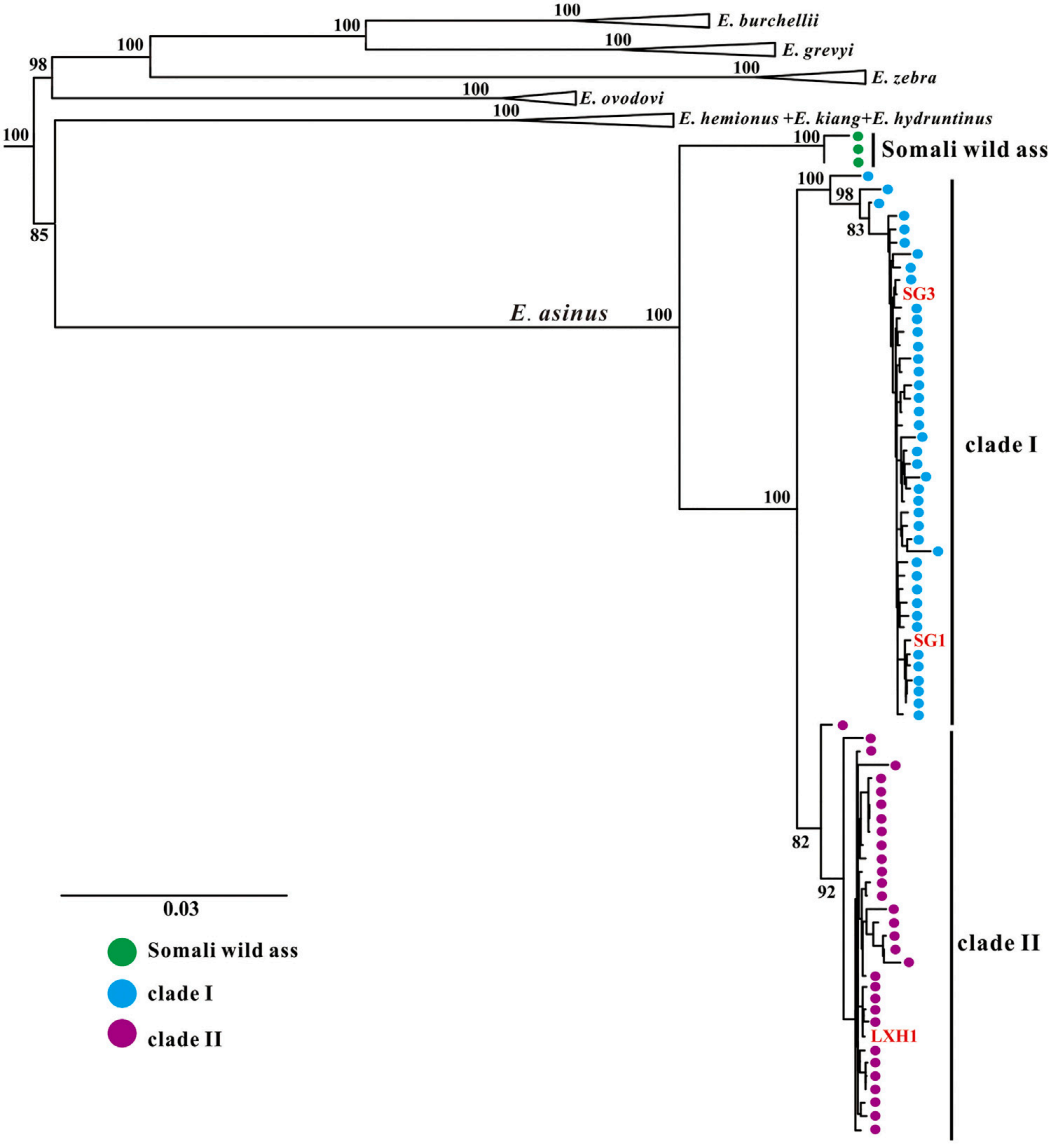
Maximum-likelihood phylogenetic tree of the genus Equus based on near-complete mitochondrial genomes. *E. caballus* was selected as outgroup (not shown here). Branch labels show bootstrap values derived from 1,000 replications.

**FIGURE 3:**
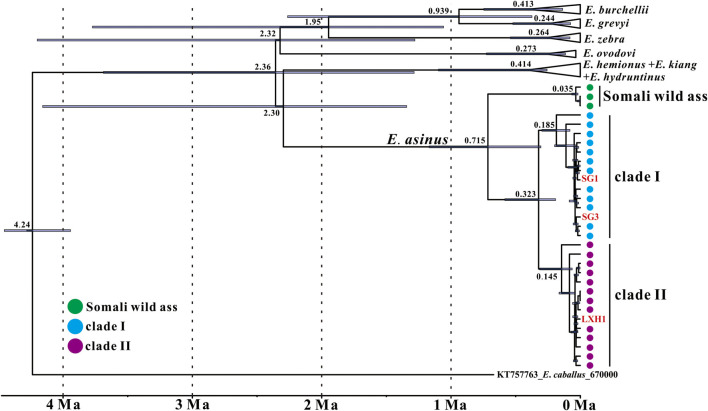
Maximum clade credibility tree of the genus Equus as recovered with BEAST based on near-complete mitochondrial genomes. Node heights are centered on the median posterior age estimates with blue bars showing 95% credibility intervals of the divergence times. Tip dates of samples used in the molecular clock analysis are listed in Supplementary Table S2.

We reconstructed phylogenetic trees using these newly obtained mitogenomes together with *Equus* sequences from GenBank. Both maximum-likelihood (ML) and Bayesian methods strongly support that all *E. asinus* individuals form a separate clade within non-caballine horses (Figures 2, 3). The *E. asinus* branch is further divided into three clades, i.e., one Somali wild ass clade, which diverges from the *E. asinus* branch first, and two domestic donkey clades (clade Ⅰ and clade Ⅱ), containing modern domesticated donkeys, Nubian wild asses and our ancient individuals (Figures 2, 3). Our results are consistent with previous studies (Kimura et al., 2010; Kimura et al., 2013; Han et al., 2014; Ma et al., 2020; Wang et al., 2020). Interestingly, the three samples investigated in this study fall into different clades of domestic donkey, i.e., specimens SG1 and SG3 cluster within clade Ⅰ (Nubian lineage), while LXH1 groups into clade II (with no extant wild representatives). Both of these two donkey clades are distant from the Asiatic wild asses (*E. kiang* and *E. hemionus*), which reveals that the maternal origin of Chinese domestic donkeys is most likely from African wild asses instead of Asian wild asses, as suggested by previous analyses (Han et al., 2014; Ma et al., 2020; Wang et al., 2020).

According to the fossil record (Epstein, 1971; Clutton-Brock, 1992; Rossel et al., 2008) and molecular data (Ivankovic et al., 2002; Beja-Pereira et al., 2004; Sun et al., 2007; Kimura et al., 2010, 2013; Han et al., 2014; Ma et al., 2020; Wang et al., 2020), African wild asses are the most likely ancestor of the domestic donkey. It is commonly believed that donkeys first dispersed from Africa to Northwest China through Central Asia about 4,000 years ago (Xie, 1987; Lu et al., 2008). If correct, this means that domestic donkeys had spread into Northwestern China before the establishment of the Han Dynasty (about the second century BC) (Han et al., 2014). After the Southern and Northern Dynasties (420-589 AD), people from Central China also started raising and breeding donkeys, and its population size gradually increased since then (Yang and Hong, 1989).

Two out of three ancient samples in this study have been dated at similar ages (i.e., 2,349-2,301 cal. BP for SG1 and 2,160-2,004 cal. BP for LXH1), yet they fall into different donkey clades (Figures 2, 3). The results demonstrate that the two donkey maternal lineages had been introduced into China at least at the beginning of Han Dynasty, i.e. around the opening of the Silk Road (about the first century BC). Unfortunately, due to a lack of earlier samples, our knowledge about when and how the two donkey maternal lineages were introduced to China is very limited so far, and further research is needed to explore these questions.

### Divergence Time of Different *E. asinus* Lineages

We carried out a mitogenome relaxed molecular clock analysis to investigate the coalescence times among *E. asinus* lineages, using root-and-tip dating calibrations in BEAST (Figure 3). Our analysis reveals that the divergence time between Somali wild ass and domestic donkey is at 0.715 Ma (95% CI: 1.169–0.305 Ma), and the split of the two domestic donkey maternal lineages is dated at 0.323 Ma (95% CI: 0.583–0.191 Ma). The times of the most recent common ancestor (TMRCA) of clade Ⅰ and clade Ⅱ are 0.185 Ma and 0.145 Ma, respectively.

Beja-Pereira et al. (2004) also estimated the divergence time of the two donkey maternal clades, and suggested a somewhat more ancient divergence in the time range of 0.910–0.303 Ma. Our point estimate (0.323 Ma) is close to the lower limit of that predicted by Beja-Pereira et al. (2004), while the confidence intervals of the two estimates overlap widely. The difference may at least partially be due to the fact that we use near-complete mitochondrial genomes to calculate the divergence time, while only cyt *b* gene sequences were included in Beja-Pereira et al. (2004). Another possible reason is that different calibration methods are implemented. Beja-Pereira *et al.* (2004) chose the previously estimated divergence time between horse and donkey (10–8 Ma) as the calibration node (Xu et al., 1996), whereas we considered the 4.5–4.0 Ma from Orlando et al. (2013) for the TMRCA of all extant *Equus* representatives and the median radiocarbon date or strata age of specimens as calibration points. Although our estimate is younger, our results together with the previous study (Beja-Pereira et al., 2004) suggest that the split of the two donkey lineages dates much earlier than its first known domestication date.

Our estimation of the TMRCA of the donkey clade Ⅰ and clade Ⅱ maternal lineages are 0.185 Ma and 0.145 Ma, respectively. This is much younger than the estimates of Kimura et al. (2010), who analyzed mitochondrial D-loop sequences of historic Nubian wild ass, Somali wild ass and ancient donkey, arriving at ages for clade I of 0.406 Ma, clade II of 0.334 Ma and the Somali wild ass clade of 0.360 Ma, respectively. However, even our younger estimates predate the domestication of donkeys by a large margin, suggesting that in both clades multiple wild lineages were incorporated into the domestic gene pool.

### Demographic Dynamics of Domestic Donkeys

**FIGURE 4:**
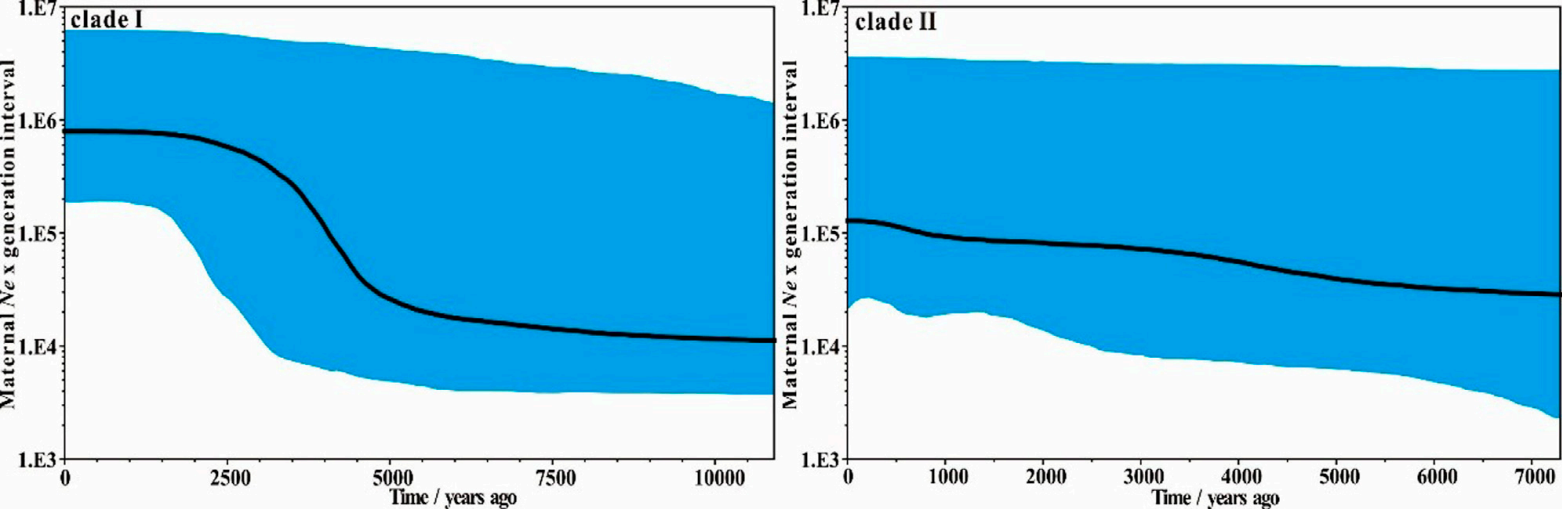
Bayesian skyline plot of the two different domestic donkey lineages based on near-complete mitochondrial genomes. Black line indicates median female *N*_*e*_ change over time, while the shaded blue area indicates the 95% credibility interval.

The Bayesian skyline plot analyses indicate an apparent population expansion between 5,000 and 2,500 years ago for clade I, following by a relatively stable population to the present day (Figure 4). However, compared to clade Ⅰ, clade II keeps a relatively stable population size overall, only showing a slight population increase during the past 5,000 years, which is similar to the result obtained by Ma et al. (2020).

Domestication of animals is generally accompanied by population expansion, as seen e.g. in horse (Fages et al., 2019), goat (Al-Araimi et al., 2017) and camel (Chen et al., 2019). The donkey population expansions of clade I and clade II may also relate to their domestication. If the Nubian lineage (clade I) and the lineage of unknown origin (clade II) were domesticated simultaneously, a similar demographic history may be expected from them. Ma et al. (2020) also assessed the population dynamics of the two domestic donkey lineages based on modern donkey mitogenomes. Their analyses suggested that clade II had a constant effective population size during most of its history, while clade I experienced a rapid population expansion starting approximately 8,000 years ago. Our estimates are overall similar to the estimates by Ma et al. (2020). In addition, Wang et al. (2020) found that there were no obvious differences in effective population size of Tropical African donkeys and North African and Eurasian donkeys, proposing that these donkeys were probably derived from the domestication of one common ancestral group. However, they noted that their analyses did not allow determining whether donkeys were domesticated at a single or multiple locations. Thus, currently, the history of donkey domestication remains at least partially unresolved. Therefore, ancient DNA is key to explore this essential question, as shown for other domesticated species such as goat (Daly et al., 2018) or cattle (Verdugo et al., 2019). Our estimates confirm that the two donkey lineages experienced somewhat different past demographic expansion histories. Together with the split time of the two clades, our results at least suggest that donkeys might have undergone at least two independent domestication events.

## Methods

### DNA Extraction

We performed DNA extraction in a dedicated ancient DNA laboratory at the University of Potsdam, following the protocol of Dabney et al. (2013) with several modifications as described in Yuan et al. (2020). The tooth samples were ground into fine powder with mortar and pestle, and for each sample about 50 mg powder was added to 1 ml extraction buffer containing 0.45 M EDTA and 0.25 mg/ ml proteinase K. The tooth powder was resuspended by vortexing and incubated overnight at 37°C under constant rotation. Next, we centrifuged the samples for 2 min at 13,300 rpm to pellet the powder, followed by adding the supernatant to 13 ml binding buffer. Then the mixtures were poured into the binding apparatus reservoirs, followed by centrifugation for 4 min at 1,500 rpm. We added 650 μL PE buffer to the silica membrane in the washing step and then carried out centrifugation again at 1,500 rpm for 4 min. DNA was eluted twice by adding 12.5 μL TET buffer each time to the silica membrane, incubating for 10 min at room temperature and centrifugation at 13,300 rpm for 30 s each time. In total, we obtained 25 μL DNA extract. In addition, an extraction blank was included alongside the samples.

### Library Construction and Hybridization Capture

Single-stranded DNA libraries were prepared by using 20 μL DNA extract for each sample, following the protocol described by Gansauge and Meyer (2013) with the modifications in Yuan et al. (2020). The amount of Circligase Ⅱ was reduced to 2 μL (100 U/ μL) in the ligation step of the first adapter; accordingly, incubation time was increased to overnight at 60°C. Hybridization capture of the complete mitochondrial genome was carried out following previously published procedures (González-Fortes and Paijmans, 2019). Baits were prepared as in the following protocol. First, total DNA was extracted from a modern horse sample and the mitochondrial genome was amplified using four overlapping long range PCR (LR-PCR) primer pairs (Vilstrup et al., 2013; Yuan et al., 2019). Second, LR-PCR products were sheared, blunt-end repaired and ligated to biotinylated adapters. Subsequently, two rounds of hybridization capture were carried out to improve the enrichment rate as described in Yuan et al. (2019). The enriched libraries were purified using Minelute columns (Qiagen) and DNA was eluted twice by adding 10 μL EB buffer each time. Concentration and fragment size of the DNA were measured on a Qubit 2.0 and a TapeStation 2200 (Agilent). Finally, the enriched libraries were pooled and sequenced on 75 cycle single-end runs on the Illumina NextSeq 500 sequencing platform, following the procedures described in Paijmans et al. (2017). Blanks were also included in single-stranded library preparation and hybridization capture procedures to monitor potential contamination.

### Data Analysis

Sequencing reads were processed as follows: 3’ adapter sequences were removed from raw reads by using cutadapt v1.4.2 (Martin, 2011), and reads shorter than 30 bp were discarded. The trimmed reads were mapped to a complete mitochondrial genome of *E. asinus* (GenBank No. X97337) using the “aln” algorithm in Burrows-Wheeler aligner (BWA) (Li and Durbin, 2010) with default parameters, and converted to bam format using the “samse” algorithm in BWA. Next, reads with a MapQuality score less than 30 and PCR duplicates were removed by using “view” and “rmdup” in samtools v0.1.9 (Li et al., 2009). Finally, a mitochondrial consensus sequence was generated in Geneious (https://www.geneious.com/), called with a minimum coverage of 2 and a base agreement greater than 75%.

### Bioinformatics Analysis

To reconstruct the phylogenetic relationships and investigate the phylogenetic status of the analyzed samples among the *E. asinus*, the three newly obtained near-complete mitochondrial genomes were aligned with 95 *Equus* mitochondrial genomes from GenBank, including *E. asinus*, *E. kiang*, *E. hemionus*, *E. hydruntinus*, *E. ovodovi*, *E. zebra*, *E. grevyi*, *E. burchellii*, and *E. caballus* using MAFFT v7.471 (Katoh et al., 2002) on the CIPRES portal (Miller et al., 2010). The ambiguous section of the D-loop was discarded and the length of the final alignment was 16,621 bp. The substitution model GTR + G for each section was selected and the data set was divided into seven partitions (Supplementary File S1) using PartitionFinder v2.1.1 (Lanfear et al., 2016). We conducted a maximum-likelihood analysis using 1,000 bootstrap replicates in RAxML-HPC v8.2.12 (Stamatakis, 2014) with *E. caballus* as outgroup.

In addition, in order to estimate the divergence time of the two main donkey maternal lineages, we also performed a Bayesian analysis in BEAST v1.8.2 (Drummond et al., 2012) using the same partitioning as above. The phylogenetic tree was calibrated by root-tip dating, using the median calibrated radiocarbon ages or stratigraphic ages for all sequences (Supplementary Table S2), and assuming a most recent common ancestor (TMRCA) of all equids of 4.0–4.5 million years ago (Ma) (Orlando et al., 2013) as calibration points. We selected constant coalescent to provide the prior distribution for the branch lengths. The GTR + G substitution model was used, running 100,000,000 generations and sampling every 10,000 steps. The first 50,000,000 samples for each chain were discarded as burn-in. The result was analyzed with Tracer v1.7 (Rambaut et al., 2018) to check effective sample size for each model parameter. A Maximum Clade Credibility tree was calculated using TreeAnnotator v1.5.4 (Drummond et al., 2012) and viewed in FigTree v1.4.4 (http://tree.bio.ed.ac.uk/software/figtree). Moreover, the donkey female effective population size changes through time (Supplementary Table S2) were estimated using the Bayesian skyline plot (BSP) analysis in Tracer v1.7 (Rambaut et al., 2018).

## Data Availability

The original contributions presented in the study are publicly available in NCBI using accession numbers MZ823384, MZ823385, and MZ823386. Our data can be freely downloaded from NCBI after October 11th 2021.
