# Supplementary material for: Ancient Mitogenomes Provide New Insights into the Origin and Early Introduction of Chinese Domestic Donkeys
Source: Front Genet. 2021 Oct 15;12:759831. doi: 10.3389/fgene.2021.759831 (PMC8554150; doi:10.3389/fgene.2021.759831)
Supplement: Supplementary file 1 [file DataSheet2.PDF]

SG1

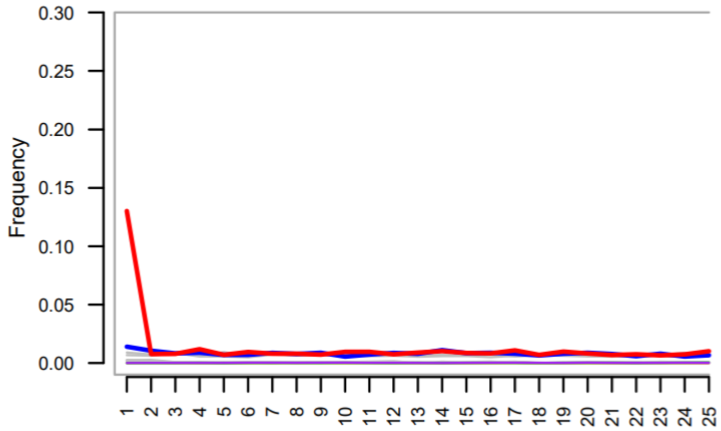

SG3

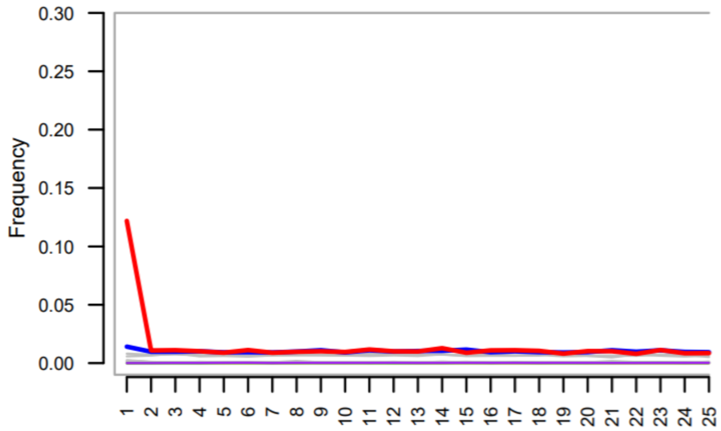

LXH1

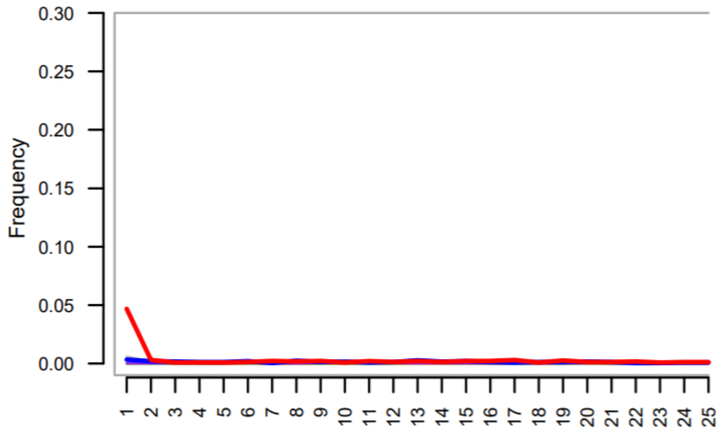

Supplementary Figure 1 Cytosine deamination frequency inferred from three donkey remains analyzed in this study. Red lines show the rates of C to T substitutions for the first 25 bases of the 5' end of the fragments that could be mapped to the donkey mitochondrial genome.
