# Supplementary material for: Ancient Mitogenomes Provide New Insights into the Origin and Early Introduction of Chinese Domestic Donkeys
Source: Front Genet. 2021 Oct 15;12:759831. doi: 10.3389/fgene.2021.759831 (PMC8554150; doi:10.3389/fgene.2021.759831)
Supplement: Supplementary file 2 [file DataSheet3.PDF]

Settings used

alignment : .\98_infile.phy
branchlengths : linked
models : GTR, GTR+G
model_selection : bic
search : greedy

Best partitioning scheme

Scheme Name : step_61
Scheme lnL : -49209.6201171875
Scheme BIC : 100868.282638
Number of params : 252
Number of sites : 16621
Number of subsets : 7

Subset	Best Model	# sites	subset id	Partition names
1	GTR+G	5431	c3cbb84f2eec1c3512017195ba06127c	_Phe, _Glu, _Asp, _16s, _Asn, _Arg, _Gly, _Ala, _ND6_CP3, _ND2_CP1, _Gln, _ND4_CP3, _ND5_CP1, _Lys, _His, _12s, _Cys, _ATP8_ATP6_CP1, _Val, _Trp, _Pro
2	GTR+G	2435	41e75ca47e23a5e06d6d0d06ee89a43a	_ND6_CP1, _Leu1, _ND4L_CP2, _CYTB_CP2, _COX2_CP1, _ND1_CP3, _ND3_CP3, _Ile, _COX1_CP2, _Met, _Ser1, _Tyr, _COX3_CP3
3	GTR+G	3611	10093ea1051617c6c45dff7d8544ad04	_ATP8_ATP6_CP2, _ND4_CP1, _ND5_CP2, _ND2_CP2, _ND4L_CP3, _COX2_CP2, _COX3_CP1, _CYTB_CP3, _COX1_CP3, _ND1_CP1, _ND3_CP1
4	GTR+G	2283	e700c08d37cdb3a372ab6896e2a7d9f5	_ND4_CP2, _ND2_CP3, _ND1_CP2, _ND5_CP3, _ND6_CP2, _CYTB_CP1
5	GTR+G	1644	9147bdb7fe421e3dd998e9c24208947c	_Leu2, _ND3_CP2, _COX1_CP1, _ND4L_CP1, _Thr, _COX2_CP3, _COX3_CP2, _ATP8_ATP6_CP3
6	GTR+G	60	c0fca4829deb62069f9e14d856d3e590	_Ser2
7	GTR+G	1157	03df7549d43f3d74a8291bb834270356	_D-loop

Scheme Description in PartitionFinder format
Scheme_step_61 = (_Phe, _Glu, _Asp, _16s, _Asn, _Arg, _Gly, _Ala, _ND6_CP3, _ND2_CP1, _Gln, _ND4_CP3, _ND5_CP1, _Lys, _His, _12s, _Cys, _ATP8_ATP6_CP1, _Val, _Trp, _Pro) (_ND6_CP1, _Leu1, _ND4L_CP2, _CYTB_CP2, _COX2_CP1, _ND1_CP3, _ND3_CP3, _Ile, _COX1_CP2, _Met, _Ser1, _Tyr, _COX3_CP3) (_ATP8_ATP6_CP2, _ND4_CP1, _ND5_CP2, _ND2_CP2, _ND4L_CP3, _COX2_CP2, _COX3_CP1, _CYTB_CP3, _COX1_CP3, _ND1_CP1, _ND3_CP1) (_ND4_CP2, _ND2_CP3, _ND1_CP2, _ND5_CP3, _ND6_CP2, _CYTB_CP1) (_Leu2, _ND3_CP2, _COX1_CP1, _ND4L_CP1, _Thr, _COX2_CP3, _COX3_CP2, _ATP8_ATP6_CP3) (_Ser2) (_D_loop);

Nexus formatted character sets
begin sets;
charset Subset1 = 1-71, 14113-14181, 6969-7043, 1113-2692, 5118-5191, 9842-9911, 9426-9496, 5044-5117, 13602-14112\3, 3936-4976\3, 3795-3864, 10212-11580\3, 11781-13601\3, 7728-7799, 11581-11649, 72-1045, 5192-5289, 7800-8642\3, 1046-1112, 4977-5043, 15398-15464;
charset Subset2 = 13604-14112\3, 2693-2767, 9913-10209\3, 14183-15325\3, 7044-7727\3, 2770-3726\3, 9499-9841\3, 3727-3794, 5358-6902\3, 3865-3935, 6903-6968, 5290-5356, 8645-9425\3;
charset Subset3 = 7801-8642\3, 10210-11580\3, 11782-13601\3, 3937-4976\3, 9914-10209\3, 7045-7727\3, 8643-9425\3, 14184-15325\3, 5359-6902\3, 2768-3726\3, 9497-9841\3;

```
	charset Subset4 = 10211-11580\3, 3938-4976\3, 2769-3726\3, 11783-13601\3, 13603-14112\3, 14182-15325\3;
	charset Subset5 = 11710-11780, 9498-9841\3, 5357-6902\3, 9912-10209\3, 15326-15397, 7046-7727\3, 8644-9425\3, 7802-8642\3;
	charset Subset6 = 11650-11709;
	charset Subset7 = 15465-16621;
	charpartition PartitionFinder = Group1:Subset1, Group2:Subset2, Group3:Subset3, Group4:Subset4, Group5:Subset5, Group6:Subset6, Group7:Subset7;
end;
```

Nexus formatted character sets for IQtree

```
#nexus
begin sets;
	charset Subset1 = 1-71, 14113-14181, 6969-7043, 1113-2692, 5118-5191, 9842-9911, 9426-9496, 5044-5117, 13602-14112\3, 3936-4976\3, 3795-3864, 10212-11580\3, 11781-13601\3, 7728-7799, 11581-11649, 72-1045, 5192-5289, 7800-8642\3, 1046-1112, 4977-5043, 15398-15464;
	charset Subset2 = 13604-14112\3, 2693-2767, 9913-10209\3, 14183-15325\3, 7044-7727\3, 2770-3726\3, 9499-9841\3, 3727-3794, 5358-6902\3, 3865-3935, 6903-6968, 5290-5356, 8645-9425\3;
	charset Subset3 = 7801-8642\3, 10210-11580\3, 11782-13601\3, 3937-4976\3, 9914-10209\3, 7045-7727\3, 8643-9425\3, 14184-15325\3, 5359-6902\3, 2768-3726\3, 9497-9841\3;
	charset Subset4 = 10211-11580\3, 3938-4976\3, 2769-3726\3, 11783-13601\3, 13603-14112\3, 14182-15325\3;
	charset Subset5 = 11710-11780, 9498-9841\3, 5357-6902\3, 9912-10209\3, 15326-15397, 7046-7727\3, 8644-9425\3, 7802-8642\3;
	charset Subset6 = 11650-11709;
	charset Subset7 = 15465-16621;
	charpartition PartitionFinder = GTR+G:Subset1, GTR+G:Subset2, GTR+G:Subset3, GTR+G:Subset4, GTR+G:Subset5, GTR+G:Subset6, GTR+G:Subset7;
end;
```

RaxML-style partition definitions

```
DNA, Subset1 = 1-71, 14113-14181, 6969-7043, 1113-2692, 5118-5191, 9842-9911, 9426-9496, 5044-5117, 13602-14112\3, 3936-4976\3, 3795-3864, 10212-11580\3, 11781-13601\3, 7728-7799, 11581-11649, 72-1045, 5192-5289, 7800-8642\3, 1046-1112, 4977-5043, 15398-15464
DNA, Subset2 = 13604-14112\3, 2693-2767, 9913-10209\3, 14183-15325\3, 7044-7727\3, 2770-3726\3, 9499-9841\3, 3727-3794, 5358-6902\3, 3865-3935, 6903-6968, 5290-5356, 8645-9425\3
DNA, Subset3 = 7801-8642\3, 10210-11580\3, 11782-13601\3, 3937-4976\3, 9914-10209\3, 7045-7727\3, 8643-9425\3, 14184-15325\3, 5359-6902\3, 2768-3726\3, 9497-9841\3
DNA, Subset4 = 10211-11580\3, 3938-4976\3, 2769-3726\3, 11783-13601\3, 13603-14112\3, 14182-15325\3
DNA, Subset5 = 11710-11780, 9498-9841\3, 5357-6902\3, 9912-10209\3, 15326-15397, 7046-7727\3, 8644-9425\3, 7802-8642\3
DNA, Subset6 = 11650-11709
DNA, Subset7 = 15465-16621
```

MrBayes block for partition definitions

```
begin mrbayes;
```

```
	charset Subset1 = 1-71, 14113-14181, 6969-7043, 1113-2692, 5118-5191, 9842-9911, 9426-9496, 5044-5117, 13602-14112\3, 3936-4976\3, 3795-3864, 10212-11580\3, 11781-13601\3, 7728-7799, 11581-11649, 72-1045, 5192-5289, 7800-8642\3, 1046-1112, 4977-5043, 15398-15464;
	charset Subset2 = 13604-14112\3, 2693-2767, 9913-10209\3, 14183-15325\3, 7044-7727\3, 2770-3726\3, 9499-9841\3, 3727-3794, 5358-6902\3, 3865-3935, 6903-6968, 5290-5356, 8645-9425\3;
	charset Subset3 = 7801-8642\3, 10210-11580\3, 11782-13601\3, 3937-4976\3, 9914-10209\3, 7045-7727\3, 8643-9425\3, 14184-15325\3, 5359-6902\3, 2768-3726\3, 9497-9841\3;
	charset Subset4 = 10211-11580\3, 3938-4976\3, 2769-3726\3, 11783-13601\3, 13603-14112\3, 14182-15325\3;
	charset Subset5 = 11710-11780, 9498-9841\3, 5357-6902\3, 9912-10209\3, 15326-15397, 7046-7727\3, 8644-9425\3, 7802-8642\3;
	charset Subset6 = 11650-11709;
			charset Subset7 = 15465-16621;


	partition PartitionFinder = 7:Subset1, Subset2, Subset3, Subset4, Subset5, Subset6, Subset7;
	set partition=PartitionFinder;

	lset applyto=(1) nst=6 rates=gamma;
	lset applyto=(2) nst=6 rates=gamma;
	lset applyto=(3) nst=6 rates=gamma;
	lset applyto=(4) nst=6 rates=gamma;
	lset applyto=(5) nst=6 rates=gamma;
	lset applyto=(6) nst=6 rates=gamma;
			lset applyto=(7) nst=6 rates=gamma;

	prset applyto=(all) ratepr=variable;
	unlink statefreq=(all) revmat=(all) shape=(all) pinvar=(all) tratio=(all);

end;
```
