# Supplementary material for: Ancient Mitogenomes Provide New Insights into the Origin and Early Introduction of Chinese Domestic Donkeys
Source: Front Genet. 2021 Oct 15;12:759831. doi: 10.3389/fgene.2021.759831 (PMC8554150; doi:10.3389/fgene.2021.759831)
Supplement: Supplementary file 3 [file DataSheet1.PDF]

Supplementary Tables

Supplementary Table 1 | Detailed information of the specimens used in this study and the obtained mitochondrial genomes.

Lab No.	Skeletal element	Location	Radiocarbon lab No.	Radiocarbon age (^{14}C, BP)	Calibrated radiocarbon age (BP)	Mitogenome reference (X97337)	
						Sequence length (bp)	Average read depth
SG1	tooth	Gaoling County, Shaanxi Province	PKU- BA170544	2,270±25	55.6% (2,349 - 2,301)	16,487	37.2
SG3	tooth	Gaoling County, Shaanxi Province	PKU- BA170545	335±20	95.4% (469 - 311)	16,531	79.5
LXH1	tooth	Linxia Basin, Gansu Province	PKU- BA172529	2,135±30	80.3% (2,160 - 2,004)	16,541	36.8

Supplementary Table 2 | Sequences downloaded from NCBI for data analyses in this study.

genus	taxon	NCBI Accession Numbers
Equus	*Equus africanus somaliensis*	AP012271_(0), KM881681_(0), MG885769_(0)
	Equus asinus (donkey: clade I lineage)	CM027722_(0), SAMN08904679_(0), SAMN08904650_(0), SAMN08904632_(0), SAMN08904613_(0), SAMN08904612_(0), SAMN08904588_(0), KT182635_(0), KX683425_(0), MG931481_(0), MK650234_(0), MK650236_(0), MK650237_(0), MK650238_(0), MK650239, MK650240_(0), MK650242_(0), MK650244_(0), MK650246_(0), MK650247_(0), MK650250, MK650251_(0), MK650252, MK650253_(0), MK650254_(0), MK650255, MK650256, MK650257_(0), MK650258_(0), MK650260_(0), MK650262_(0), MK650264_(0), MK650267_(0), MK650268, MK896291_(0), MK896292, MK896296_(0), MK896300_(0), MK896305_(0), MK896307_(0), MK982180_(0)
	Equus asinus (donkey: clade II lineage)	SAMN08904683_(0), SAMN08904634_(0), SAMN08904626_(0), KX669267_(0), MK650269_(0), MK650271_(0), MK650272_(0), MK650274_(0), MK650275_(0), MK650276_(0), MK650277_(0), MK650278_(0), MK650279_(0), MK650280_(0), MK650282_(0), MK650283_(0), MK650284_(0), MK650285_(0), MK650286_(0), MK896295_(0), MK896297_(0), MK896298_(0), MK896299_(0), MK896301_(0), MK896302_(0), MK896303_(0), MK896304_(0), MK896306_(0), MK896308_(0), NC_001788_(0), X97337_(0)
	Equus burchellii	KM881680_(0), JX312733_(0), NC_018781
	Equus grevyi	JX312720_(0), JX312722_(0), JX312725
	Equus zebra	JX312717_(0), NC_020476_(0)
	Equus ovodovi	JX312734_(40000), KY114520_(34307), PRJNA511614, PRJNA512859, PRJNA512861
	Equus hemionus	HM118851, JX312728_(0), JX312730_(0)
	Equus kiang	JX312731_(0), JX312732_(0)
	Equus hydruntinus	MK574675_(22000)
	Equus caballus	KT757763_(670000)

Note: Ages used in BEAST analysis are indicated in parentheses following the accession number.
